# Diversity of Dominant Peripheral T Cell Receptor Clone and Soluble Immune Checkpoint Proteins Associated With Clinical Outcomes Following Immune Checkpoint Inhibitor Treatment in Advanced Cancers

**DOI:** 10.3389/fimmu.2021.649343

**Published:** 2021-06-07

**Authors:** Ye Li, Jiaqian Wang, Liangliang Wu, Xiaoting Li, Xiaoyun Zhang, Guoqing Zhang, Shengqiang Xu, Shengjie Sun, Shunchang Jiao

**Affiliations:** ^1^ Department of Radiotherapy, First Medical Center of Chinese PLA General Hospital, Beijing, China; ^2^ Oncology Laboratory, First Medical Center of Chinese PLA General Hospital, Beijing, China; ^3^ Medical School of Chinese PLA, Beijing, China; ^4^ YuceBio, Shenzhen, China; ^5^ YutaiAntigen, Shenzhen, China; ^6^ Department of Oncology, First Medical Center of Chinese PLA General Hospital, Beijing, China

**Keywords:** advanced cancer, immune checkpoint inhibitors, T cell repertoire, high-throughput sequencing, soluble checkpoint protein

## Abstract

Dynamic changes of the peripheral T cell receptor (TCR) and soluble receptors and ligands (sRLs) have the potential to be used as biomarkers to monitor the evolution of the immune system in tumor patients undergoing immunotherapy. These functional biomarkers could be used to predict immune response to treatment with immune checkpoint inhibitors (ICIs) and to provide high-value information on the immune function status of cancer patients, thereby helping physicians to make effective clinical decisions. We collected paired pre- and post-treatment peripheral blood samples from 31 solid tumor patients treated with ICIs. TCR and sRL status were investigated using next-generation sequencing and magnetic bead panels. We found that the diversity of the dominant TCR clone at baseline was correlated with durable clinical benefit in patients receiving single-agent treatment. The D50 index, the diversity from the cumulative 50% of the total complementary determinant region 3, was obtained during treatment. A significant difference in progression-free survival was demonstrated between the D50 high and D50 low groups. This result was validated in an independent cohort. A signature including soluble immune checkpoint proteins (sICPs) was identified. Upregulation of the signature during treatment was correlated with durable clinical benefit. All these results indicate that a novel biomarker based on peripheral TCR and sICPs has the potential to be used in prognostic prediction and for rapid determination of therapeutic outcomes in patients treated with immune checkpoint inhibitors.

## Introduction

In recent years, immune checkpoint inhibitors (ICIs) that target cytotoxic T lymphocyte-associated antigen (CTLA-4), programmed cell death receptor-1 (PD-1), and programmed death ligand-1 (PD-L1) have led to revolutionary advances in tumor immunotherapy (1). However, only a minority of patients achieve clinical benefit from ICI therapy, and there remain many challenges when using current biomarkers to predict the efficacy of ICI therapy. Although certain biomarkers have been approved by the Food and Drug Administration for various tumors, the quantity and quality of biopsies are not consistent. Moreover, biomarkers for in-treatment evaluation are useful, especially those obtained from non-invasive material such as peripheral blood. Biomarkers from peripheral blood can provide real-time information about the patient’s immune system.

The peripheral T cell receptor (TCR) repertoire reflects the immunological status of all functional T cells in an individual’s circulatory system at a given time. Each unique T cell has a unique TCR, which varies among individuals within a population. The complementary determinant region 3 (CDR3), encoded by random rearrangements and junctions of variable, diversity, and joining [V(D)J] sequences, plays an important part in the recognition of tumor antigens (2) and contributes to the diversity of the TCR. The diversity of the TCR determines the immune effects against pathogens. Currently, next-generation sequencing-based technologies are widely employed for high-throughput analysis of the immune cell repertoire. The development of biomarkers based on TCR-CDR3 sequencing will be meaningful for cancer therapeutic decision-making (3).

Soluble receptors and ligands (sRLs) are important components of immune regulation. Their expression levels in serum can be detected. Some sRLs have already been used when assessing clinical severity and prognosis in cancer patients (4, 5). Soluble cytokine receptors (sCKR) include proteolytic cleaved cell-surface receptors, extracellular release of membrane-bound receptors, and alternative gene-generated cytokine-binding proteins. They are key regulators of inflammation and immune responses (6, 7). Immune checkpoints regulate immune response and the proteins expressed by immune cells or tumor cells. They have an important role in preventing excessive immune response and maintaining normal immune homeostasis, and can also help tumor cells to escape attack by T cells. The application of soluble immune checkpoint proteins (sICPs) has been studied for its potential to predict the immunotherapy response.

In this study, we performed peripheral TCR sequencing and sRL magnetic bead panel testing using samples from tumor patients who had been treated with PD-1 or PD-L1 ICIs. A novel biomarker was discovered and validated in clinical cohorts. The biomarker shows promise for use in clinical practice owing to its rapid and non-invasive application.

## Materials and Methods

### Patients and Sample Collection

A total of 31 patients with advanced solid tumors who were treated in the Oncology Department of the General Hospital of the Chinese People’s Liberation Army from January 2016 to September 2018 and received ICIs were enrolled in this research. Informed consent forms were obtained from all participants. All patients with solid tumors received intravenous injections of either PD-1 or PD-L1 inhibitors, including nivolumab, pembrolizumab, atezolizumab, durvalumab, and sintilimab. Patients were treated with or without combined chemotherapy or targeted therapies until the tumor progressed or intolerable adverse reactions occurred. Clinical hematology, bone marrow cell morphology, histochemical staining, immunology, molecular biology examination, and the FAB collaborative group diagnostic criteria were used to confirm that the patients did not have any infectious disease, autoimmune disease, or other immune-related diseases. Blood samples were obtained at baseline before treatment with anti-PD-1/PD-L1; one additional blood sample was obtained after treatment to assess dynamic changes in 24 patients. Peripheral blood mononuclear cells (PBMCs) were isolated, added to a protective solution, and immediately stored at -80°C until further processing. Patients’ clinical data were collected prospectively. Disease progression was evaluated based on version 1.1 of the RECIST criteria. The study was conducted according to the Helsinki guidelines and was approved by the Ethics Committee of the General Hospital of the People’s Liberation Army (S2016-097-01); written informed patient consent was obtained.

### High-Throughput Sequencing of TCR

PBMCs were isolated by standard Ficoll gradient separation from whole blood within 4 h of collection. Lamina propria mononuclear cells were isolated from endoscopic biopsies. Total RNA was extracted using TRIzol reagent (Invitrogen, USA) according to the manufacturer’s instructions, and the final concentration was determined using a NanoDrop 2000 spectrophotometer (Life Technologies). Then, 1 µg of total RNA was used for cDNA synthesis with random hexamers using a Qiagen One-step RT PCR kit (Qiagen, USA). The CDR3 region of the TCR β chain was amplified using the iRepertoire multiplex primer set (iRepertoire, Inc.). A set of nested primers specific to various V and constant (C) elements of the TCR β chain were used in the first amplification cycles, followed by amplification using communal primers based on the manufacturer’s protocol. Libraries were purified by agarose gel electrophoresis, cut between 200–400 bp (predicted amplicon size 210–310 bp), extracted (Qiagen), and sequenced (Illumina 4000, Illumina Inc.). The read length of sequencing was 150 bp. The sequencing technology platform used in our study was proven to be feasible and repeatable by a technical duplicate test.

### TCR Sequence Analysis

Sequence reads were de-multiplexed according to barcode sequences at the 5′ ends of reads from the TCR β constant region. Reads were then trimmed according to their base quality to remove low-quality 3′ ends [FastX-toolkit (8), Cutadapt, SOAPnuke (9)]. Trimmed pair-end reads were joined together through overlapping alignment with a modified Needleman–Wunsch algorithm. If paired forward and reverse reads in the overlapping region were not perfectly matched, both forward and reverse reads were discarded without further consideration. The merged reads were mapped by use of an edited version of MiXCR (10) to identify V and J genes across the germline V, D, J, and C reference sequences downloaded from the IMGT (11) website. To define the CDR3 region, the positions of CDR3 boundaries of reference sequences from the IMGT database were migrated onto reads through mapping results, and the resulting CDR3 regions were extracted and translated into amino acid sequences. Then, aligned reads were aggregated into clonotypes based on the CDR3 nucleotide sequence and the same IGH V(D)J gene segment using open source software VDJtools (https://github.com/mikessh/vdjtools ) version 1.1.1. A frequency-based correction was performed with default parameters. Corrected samples were stored as clonotype abundance tables for subsequent analyses. Figures including heatmaps and plots of V gene usage frequencies were generated using the programming language R. The Shannon–Wiener index (12) was used to identify the diversity of the TCR receptor. The D50 index was used as a measure of the diversity from the cumulative 50% of the total CDR3 counted in the sample (13).

### sCKR and Checkpoint Protein Measurements

The concentrations of human sCKRs (sCD30, sEGFR, sgp130, sIL-1RI, sIL-1RII, sIL-2Rα, sIL-4R, sIL-6R, sRAGE, sTNFRI, sTNFRII, sVEGFR1, sVEGFR2, sVEGFR3) and the Human Immuno-Oncology Checkpoint Protein panel (sBTLA, sCD27, sCD28, sTIM-3, sHVEM, sCD40, sGITR, sLAG-3, sTLR-2, sGITRL, sPD-1, sCTLA-4, sCD80, sCD86, sPD-L1, sICOS) in plasma and sputum samples were determined using Milliplex MAP Multiplex kits (EMD Millipore, Billerica, MA) according to the manufacturer’s recommended protocols. Following thawing, plasma and processed sputum samples were centrifuged at 10,000×g for 5 min, and the supernatants were assayed according to the manufacturer’s instructions. All primary data points were collected on a Luminex FLEXMAP 3D^®^ system with concentrations calculated based on seven-point standard curves (*R* ^2^ > 0.95) using a five-parametric fit algorithm in xPONENT^®^ v4.0.3 (Luminex Corp., Austin, TX). Only data that fell within the documented assay range, passed the quality control/assurance measures provided by each kit’s manufacturer, and possessed a %CV (Coefficient of Variation) value ≤10% were considered by our statistician. Any primary read that failed to meet these quality control thresholds was reprocessed until a satisfactory value was obtained. The standard curve for each analysis had an *R* ^2^ value >0.95 with or without minor fitting using xPONENT^®^ software.

### Statistical Analysis

All statistical analyses were performed using R software. The significance of differences between groups was estimated by paired, two-tailed student’s t-test, Fisher’s exact test, or Mann–Whitney test, as appropriate; p-values less than 0.05 were considered to indicate statistical significance.

## Results

### Patient Characteristics

A total of 31 solid tumor patients treated with anti-PD-1/PD-L1 were enrolled in this study (**Table 1**). There were five tumor types, comprising non-small-cell lung carcinoma (NSCLC; 21 cases), urothelial carcinoma (five cases), gastric adenocarcinoma (two cases), hepatocellular carcinoma (two cases), and intrahepatic cholangiocarcinoma (one case). Fourteen (45.16%) patients were treated with a single anti-PD-1/PD-L1 agent, and 17 (54.84%) were treated with a combination of chemotherapy or targeted therapies and anti-PD-1/PD-L1. The ICI drugs used included pembrolizumab (42%), nivolumab (42%), atezolizumab (10%), durvalumab (3%), and sintilimab (3%). Using version 1.1 of the RECIST criteria to evaluate progression, a total of 15 (48%) patients were assessed as SD (stable disease), nine (29%) patients as PD (progressive disease), and seven (23%) patients as PR (partial response). The PR and SD patients surviving more than six months were assigned to the durable clinical benefit (DCB) group (35%); the others formed the non-durable benefit (NDB) group (65%). Patients’ clinical data are shown in **Supplementary Table 1**.

**Table 1:** The demographic and clinical characteristics of 31 patients with anti-PD-1/PD-L1 treatment.

	Characteristics	Number of cases
Age	<40	7 (23%)
	>60	13 (42%)
	40-60	11 (35%)
Gender	Female	10 (33%)
	Male	21 (67%)
Smoking	No	17 (55%)
	Yes	13 (42%)
	Unknown	1 (3%)
Alcohol	No	19 (62%)
	Yes	11(35%)
	Unknown	1 (3%)
Immune checkpoint blockade therapy	Single-agent	14 (45%)
	Combination	17 (55%)
Cancer type	Non-small-cell lung carcinoma	21 (68%)
	Urothelial carcinoma	5 (16%)
	Gastric carcinoma	2 (6%)
	Hepatocellular carcinoma	2 (6%)
	Intrahepatic cholangiocarcinoma	1 (3%)
Clinical benefit	DCB	11 (35%)
	NDB	20 (65%)

### Correlation Between TCR Repertoire and Clinical Efficacy at Baseline

**Figure 1:**
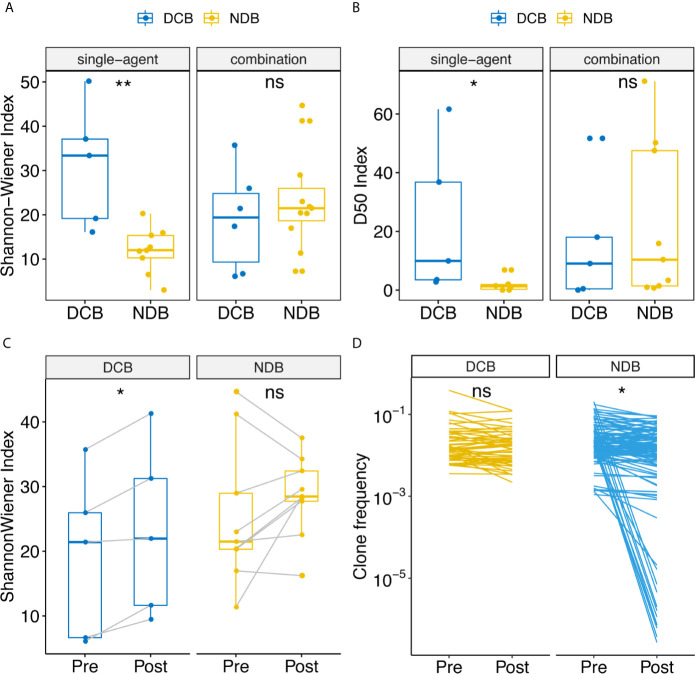
Comparison of diversity of TCR repertoire at baseline between clinical benefit and non-clinical benefit patients with advanced tumors treated with anti-PD-1/PD-L1 and dynamic change of TCR repertoire at pre- and post-treatment samples correlated with clinical benefit. Shannon-Wiener and D50 index diversity were compared between clinical benefit (blue points) and non-clinical benefit (yellow points) patients. Statistical analysis was performed using the Mann-Whitney test. Boxes depict the interquartile range with the line in boxes showing the median, and the lines outside the boxes showing the first or third quartiles of fraction. ns p >= 0.05, *p < 0.05, **p < 0.01. **(A)** Shannon-Wiener index distribution in single-agent patients and combination patients. **(B)** D50 index distribution in single-agent patients and combination patients. **(C)** Dynamic change of Shannon-Wiener index distribution in patients treated with combination. **(D)** Clonotype frequencies that exist at 0.1% at baseline for clinical benefit and non-clinical benefit patients.

The Shannon–Wiener index (12) is widely used to measure TCR receptor diversity. Previous studies have reported that clones with high frequency are associated with response to immunotherapy at baseline (14). Therefore, we calculated the Shannon–Wiener index in TCR clones with frequency higher than 0.1% and 1%. Association analysis showed that the Shannon–Wiener index of clones with frequency higher than 0.1% was associated with durable response only in the patient group treated with single-agent anti-PD-1/PD-L1 (**Figure 1A**). The median Shannon–Wiener index in the DCB group was 33.37, which was 2.78 times that in the NDB group (p = 0.0040). It is noteworthy that no significant difference was found in the combination group. This was probably because the mechanism of combination therapy was more complex, enabling it to improve the microenvironment of tumors through various mechanisms and change the diversity of T cells more in the course of treatment so as to benefit clinically. Therefore, the diversity of TCR at the baseline level cannot effectively reflect the clinical efficacy of combination therapy. Another notable thing is that the diversity measurement was only found significant in the TCR clone with frequency higher than 0.1%. These indicated that the dominant clone rather than the global clone accounted for the response of anti-PD-1/PD-L1. We then used the D10, D20, D50 indexes (13), which are defined as the minimum percentage of distinct CDR3s accounting for at least 10%, 20%, or 50% of the total CDR3s in a population of T cells to measure diversity, because these indexes focused on the diversity calculation of dominant unique clones. Significant differences in the D50 index were found between the DCB and NDB groups: the D50 index was 4.69 times higher in the DCB group than in the NDB group (p = 0.022, **Figure 1B** and **Supplementary Figures 1A, B**).

Comparative analysis of Vβ, Jβ, and Vβ-Jβ paired gene usage in TCR repertoires at baseline between the DCB and NDB groups was performed to further investigate the preferential usage of anti-PD-1/PD-L1 treatment. As shown in **Supplementary Figures 1C, D**, the usage of the V gene and J gene had relatively similar frequency between the two groups, but only three fragments were significantly differentially expressed between the two groups. TRBV15 fragments had higher frequency in the DCB than the NDB group (p < 0.05), whereas TRJ1-3 fragments in the J gene had lower frequency (p < 0.05). These three significantly differentially expressed fragments were abundantly expressed in tumor patients, and their differential expression indicated that the two clinical benefit groups had different preferential usage of the TCR Vβ and Jβ genes.

### Dynamic Change of the TCR Repertoire Is Correlated With Clinical Characteristics

In order to investigate whether treatment with ICIs caused global T cell expansion and/or loss of T cell clonotype diversity in patients, and whether the dynamic changes of the TCR receptor could be used as a marker for predicting clinical efficacy, we collected one additional blood sample from each patient after treatment. A total of 24 samples were collected and prepared for TCR sequencing. The additional time point during therapy was at a median of nine weeks (from 3 to 36 weeks). Pearson’s correlation coefficient was used to define the similarity of the diversity of the TCR receptor pre- and post-treatment. The similarity was found to be related to time and therapy (**Supplementary Figures 2A, B**). The longer the therapy time, the lower the similarity, and the greater the change in TCR diversity (p = 0.0076). The correlation coefficient was higher for single-agent than for combination therapy (p = 0.043), confirming the previous hypothesis that combination therapy might lead to greater changes in the global TCR repertoire.

Considering that the TCR repertoire of samples changed greatly with the time of treatment, we used blood samples taken within nine weeks from the start of treatment to analyze the correlation with clinical efficacy. The Jaccard index (14), which was used to define the similarity of TCR clones, was significantly different between the DCB and NDB groups (p = 0.031); the lower the Jaccard index, the better the clinical efficacy, especially in the combined therapy sample (**Supplementary Figure 2C**). The Jaccard index was used to compare TCR similarity by determining which clones were present in both pre- and post-treatment samples. Thus, the results suggest that anti-PD-1/PD-L1 treatment affected the changes to the TCR repertoire, and that the degree of TCR change reflected the clinical response.

To assess whether the dynamic changes of the Shannon–Wiener index between pre- and post-treatment were related to clinical outcomes, we performed a correlation analysis. In the combination group, the Shannon–Wiener index was significantly increased in DCB patients, with an average increase of 20.44% (**Figure 1C**, p = 0.031), whereas there was no significant change in NDB patients. This suggests that upregulation of TCR diversity by 20.44% would indicate DCB. To understand which type of TCR clones were changed, we first focused on the top 10 high-expressed TCR clones (15). The expression abundance of high-frequency TCR clones in baseline and post-treatment samples decreased significantly in the NDB group (**Figure 1D**, p < 0.001) but not in the clinical benefit group. This indicates that the high-frequency expression of TCR clones was maintained in the clinical benefit group.

**Figure 2:**
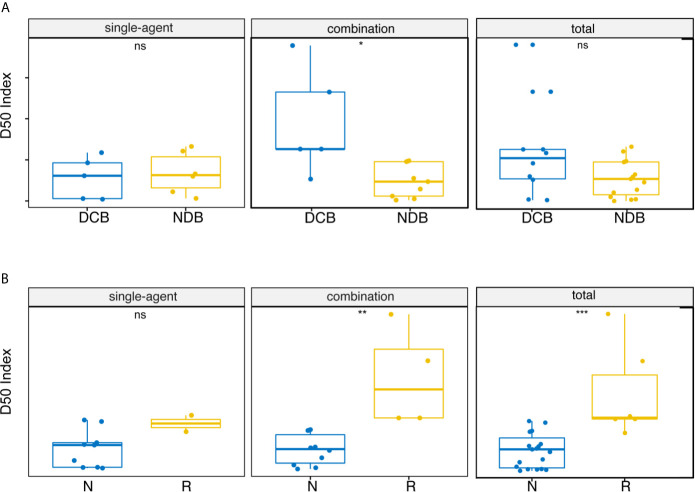
Correlations between D50 index and clinical benefit. **(A)** Correlations between D50 index and durable clinical benefit at post-treatment samples. **(B)** Correlations between D50 index and response at post-treatment samples. Statistical analysis was performed using the Mann-Whitney test. Boxes depict the interquartile range with the line in boxes showing the median, and the lines outside the boxes showing the first or third quartiles of fraction. ns p >= 0.05, *p < 0.05, **p < 0.01, ***p < 0.001.

**Figure 3:**
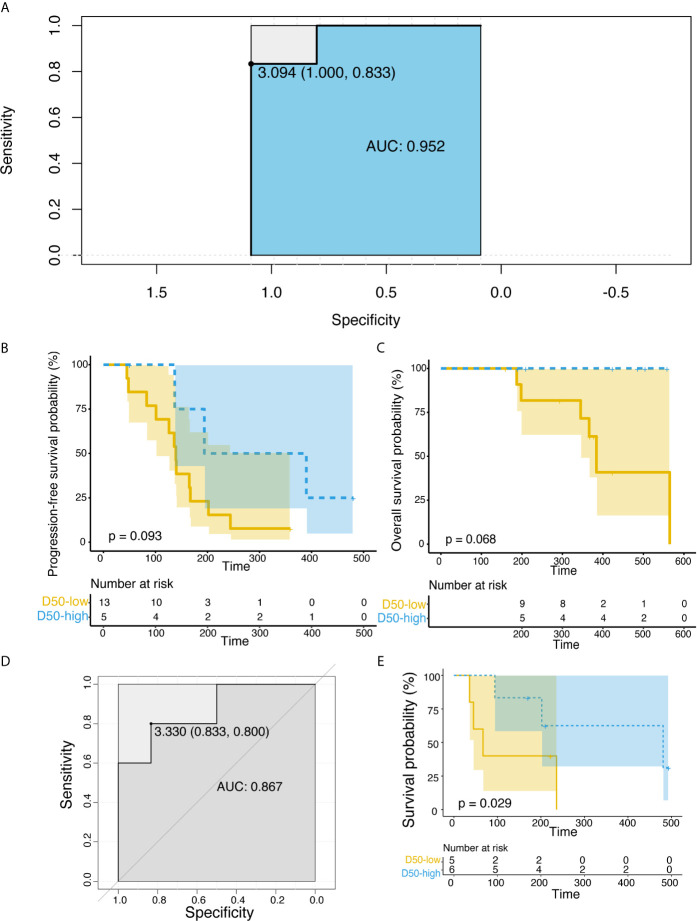
Correlations between D50 index and progression-free survival rate in the discovery and validation cohorts. **(A)** ROC curve for the correlation of D50 index with response in the discovery cohort. AUC is 0.952. Cut-off of ≥3.09 D50 index is designated by triangle. **(B)** Correlations between D50 index and progression-free survival rate at post-treatment samples in the discovery cohort. **(C)** Correlations between D50 index and overall survival rate at post-treatment samples in the discovery cohort. **(D)** ROC curve for the correlation of D50 index with response in the validation cohort. **(E)** Correlations between D50 index and progression-free survival rate at post-treatment samples in the validation cohort. The survival curves were constructed using the Kaplan-Meier method and compared between groups classified by the D50 index in **(B**, **D)**. Significance was analyzed by log-rank test.

We inferred that the diversity of high-frequency TCR expression in post-treatment samples might be related to clinical efficacy. Therefore, we expanded the dominant T cell clones that accounted for the cumulative 50% of the total CDR3 counted in the sample and used the D50 index to measure the diversity of samples after treatment. Significant correlation between the D50 index and DCB after treatment was found only in the combined therapy group (**Figure 2A**, p = 0.012). The D50 index was also associated with response in both the combined therapy group (**Figure 2B**, p = 0.0020) and the all patients group (**Figure 2B**, p = 0.00021). The higher the D50 index, the more likely patients were to show response. The D50 index was also higher in responders than non-responders in the single-agent group, but this difference did not reach statistical significance. The area under the curve (AUC) of the receiver operating characteristic (ROC) curve was 0.952 when predicting the response of objective response rate (ORR) (**Figure 3A**). The sensitivity and specificity of the response using a D50 of 3.09 as a cut-off point were 100% and 83.30%, respectively. Patients were divided into D50 high and low groups based on the Youden index (16) (the maximum sensitivity and the best specificity under the ROC curve). A significant difference in progression-free survival (PFS) rate was identified between the D50 high and D50 low groups (**Figure 3B**, p = 0.098). Overall survival was also longer in D50 high patients than in D50 low patients (**Figure 3C**, p = 0.068).

To validate the potential of the D50 index for prognostic prediction, an additional validation cohort of 11 NSCLC patients treated with anti-PD-1/PD-L1 was established (**Supplementary Table 3**). The D50 index was higher in DCB patients (**Figure 3D**), and the PFS rate was significantly greater in D50 high patients (log-rank p = 0.029) (**Figure 3E**). We further analyzed at which time points the D50 index could predict response. By comparing the D50 index at different blood collection points with the clinical benefit group, we observed that the D50 index could significantly differentiate DCB patients at six weeks (**Supplementary Figure 3A**, p = 0.0088).

We continued to identify TCR clones with significant differences at baseline and post-treatment. Briefly, Fisher’s exact test was used to generate a p-value for every T cell clone, and p-values were adjusted using a positive false discovery rate method to identify significantly expanded T cell clones. Similarly, the number of differentially expressed TCR clones was correlated with clinical efficacy. The number of differentially expressed TCR clones in the clinical benefit group was higher than that in the non-clinical benefit group (median 27 versus 18, p = 0.038, **Supplementary Figures 3B, C**). We focused on clonal expansion of TCR clones at baseline and found three shared TCR clones (**Supplementary Table 2**). These clones were expanded in at least two samples, and most in DCB groups, suggesting that they indicated an immune-selective response. Harnessing these T cells could provide practical strategies to improve the shared antigen-specific response to cancer.

### Peripheral sRL Landscape and Therapeutic Outcomes

The human immune response can be regulated by sRLs, including sCKRs and sICPs. Monitoring the expression levels of sRLs in peripheral blood could help us to understand the immune function of the body; this could be of use in monitoring the treatment of disease and evaluating prognosis, as well as providing a reference for clinical treatment. In order to understand patients’ clinical response to ICI treatment, we analyzed sRLs in 24 paired pre- and post-treatment samples. The sCKRs included sCD30, sEGFR, sgp130, sIL-1RI, sIL-1RII, sIL-2Ra, sIL-4R, sIL-6R, sRAGE, sTNFRI, sTNFRII, sVEGFR1, sVEGFR2, and sVEGFR3. The sICPs included sBTLA, sCD27, sCD28, sTIM-3, sHVEM, sCD40, sGITR, sLAG-3, sTLR-2, sGITRL, sPD-1, sCTLA-4, sCD80, sCD86, sPD-L1, and sICOS.

**Figure 4:**
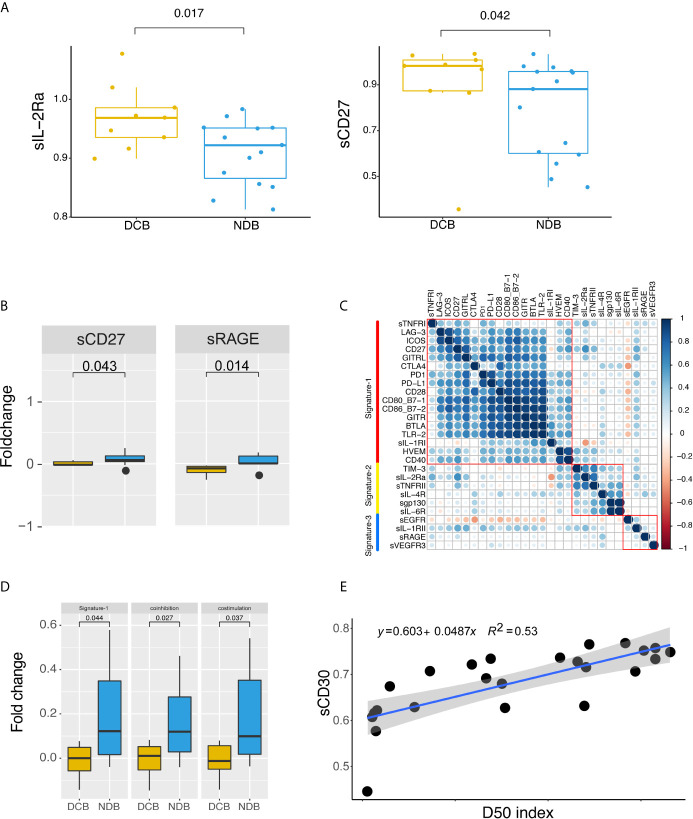
Peripheral soluble receptors and ligands landscape and therapeutic outcome. **(A)** Different expression of soluble receptors and ligands between clinical benefit groups at baseline. **(B)** Dynamic changes of sCD27 and sRAGE compared between clinical benefit groups. **(C)** Correlation map of dynamic changes in soluble cytokine receptors and immune checkpoint protein expression. Correlations were defined using paired person correlation. **(D)** Signature-1 genes were differently expressed between clinical benefit groups. **(E)** Correlation between D50 index and sCD30. Statistical analysis of **(A, B, D)** was performed using the Mann-Whitney test. Boxes depict the interquartile range with the line in boxes showing the median, and the lines outside the boxes showing the first or third quartiles of fraction.

We first evaluated differentially expressed sRLs in baseline samples. As shown in **Figure 4A**, there were significant differences in the expression of sIL-2Ra and sCD27 between the DCB group and the NDB group (p < 0.05). Expression levels of sIL-2Ra and sCD27 were more than 5.04% and 11.52% higher in the DCB group than in the NDB group, respectively.

In order to better understand the dynamic changes between baseline and post-treatment, we performed a fold-change comparison for all sRLs between baseline and post-treatment. When considering single sCKRs or sICPs, as shown in **Figure 4B**, sRAGE and sCD27 showed significant fold changes before and after immunotherapy (p < 0.05); both were upregulated in post-treatment samples. We then analyzed whether signatures of fold change were associated with clinical efficacy. The correlation results for the fold changes of each sRL are shown in **Figure 4C**. Three fold change signatures were identified. Notably, Signature-1 included sCD27, sBTLA, sCD28, sCD40, sGITR, sLAG-3, sTLR-2, sGITRL, sPD-1, sCTLA4, sCD80, sCD86, sPD-L1, sICOS, sTNFRI, sIL-1RI, and sHVEM, that is, most of the immune checkpoint proteins. We normalized the signatures by taking the average expression levels of the signature genes for each patient, and then analyzed the relationship between the signatures and clinical benefit. As shown in **Figure 4D**, Signature-1 was significantly upregulated in the NDB group compared with the DCB group (p < 0.05). Signature-1 mostly contained immunological checkpoint proteins. We further subdivided the Signature-1 genes into a co-inhibitory protein group and a co-stimulatory protein group. There were significant differences in co-stimulatory proteins and co-inhibitory proteins between the DCB and NDB groups, with higher levels of co-stimulatory and co-repressor proteins in the NDB group. These results indicate that upregulation of sICP might have adverse clinical effects.

We divided the patients into two groups according to upregulation and downregulation of Signature-1 genes, and compared the TCR diversity between the two groups. Patients with upregulation of Signature-1 showed more downregulation of TCR clones from baseline to post-treatment (p = 0.0085, **Supplementary Figure 4A**), indicating a correlation with TCR diversity. Finally, we analyzed the association of D50 with sCKRs and sICPs, and found that the D50 index was significantly associated with sCD30 (p = 0.000032, **Figure 4E**).

**Figure 5:**
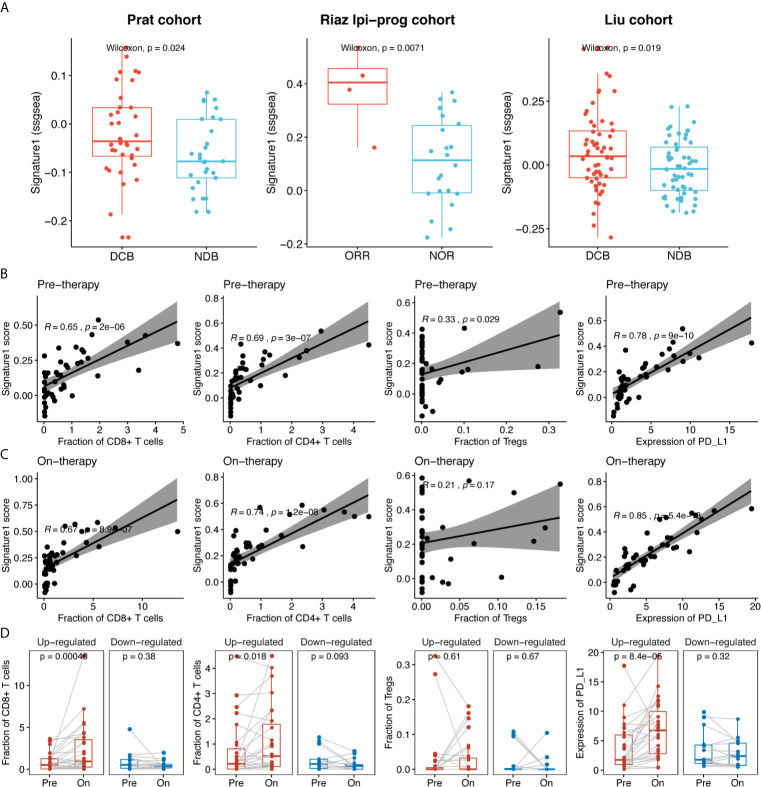
The clinical efficacy of Signature-1 validated in three published cohorts**. (A)** Correlations between Signature-1 score (used “ssgsea” method in GSVA R package) and durable clinical benefit in Prat et al., Riaz et al., and Liu et al. **(B, C)** Correlations between the fraction of CD8+ T cells, CD4+ T cells, Tregs, and PD-L1 expression level and Signature-1 in pre-therapy (baseline) and on-therapy samples. **(D)** Dynamic change of the fraction of CD8+ T cells, CD4+ T cells, Tregs, and PD-L1 expression level between pre-therapy (baseline) and on-therapy samples in Signature-1 upregulated and downregulated groups. Upregulated: Log2 fold change of Signature-1 score in on-therapy and pre-therapy samples > 0; Downregulated: Log2 fold change of Signature-1 score in on-therapy and pre-therapy samples < 0.

To further validate the association of the sICP signature with clinical efficacy, we used RNA sequencing data of immunotherapy cohorts from Prat et al. (17), Riaz et al. (18), and Liu et al. (19), and calculated Signature-1 scores based on RNA expression. Signature-1 scores were significantly higher in the DCB group than in the NDB group in the Prat, Riaz Ipi-prog (ipilimumab-progressed), and Liu cohorts (p = 0.024, p = 0.0071, and p = 0.019, respectively, **Figure 5A**). In order to explore the correlation between Signature-1 and the intratumoral immune compartment, we used Riaz’s tumor RNA data to evaluate the correlations among immune cells, PD-L1, and Signature-1. For both pre- and post-treatment samples, Signature-1 showed significant correlations with CD8+ and CD4+ T cells and PD-L1 expression (**Figures 5B, C**). The fractions of CD8+ and CD4+ T cells and PD-L1 in the Signature-1 upregulated group were increased significantly (**Figure 5D**). These results further validate the prediction ability of the gene set from Signature-1.

We also performed a correlation analysis between TCR diversity and immune cells. The results were similar to those described above: TCR diversity was more correlated with CD8 and CD4 T cells. There was also a certain correlation with the expression level of PD-L1 (**Supplementary Figure 5**).

## Discussion

It is not easy to obtain tumor tissues from patients with advanced disease in order to dynamically monitor the efficacy of immunotherapy. Therefore, it is of great value to develop peripheral blood biomarkers to predict the clinical benefit of anti-PD-1/PD-L1 therapy. In this study, 31 patients with baseline data were enrolled; for 24 patients, paired blood samples were also collected during treatment to explore clinical biomarkers. This is the first systematic exploration of peripheral blood markers in patients treated with ICIs. The TCR repertoire, sCKRs, and sICPs in peripheral blood were analyzed at the same time.

We first observed that the diversity of the TCR dominant clone at baseline was associated with DCB in patients receiving single-agent therapy. The greater the diversity, the more likely the patients were to show durable benefit. However, we did not observe any such correlation in patients receiving combination therapies. Most of the patients in our study were treated with anti-PD-1/PD-L1 combined with chemotherapy. Chemotherapy can induce immunogenic death of tumor cells, release tumor antigens, and activate more T cells that recognize tumors. This was confirmed by the comparison analysis of the dynamic changes of TCR for the two therapies. That is, the changes in TCR clones were greater in patients treated with combination therapy. Therefore, TCR diversity at baseline was not correlated with durable benefits in the combination cohort. Furthermore, we found that the dominant clone (that occupying >0.1% of the repertoire) was associated with DCB. The dominant clone was also found to be significantly associated with ICI response in a recent study of metastatic melanoma (20).

When comparing dynamic changes of TCR diversity between pre- and post-treatment samples, we found that changes in TCR diversity were related to treatment time and treatment method. The diversity in DCB patients with combination therapy increased significantly (i.e., the Shannon–Weiner index increased by 20.44%), but that in NDB patients did not. This indicates that the diversity of TCR generally increased in the DCB group. We then analyzed which aspect of TCR clone abundance was beneficial with respect to DCB. The results showed that the highly expressed TCR clone remained highly expressed in the clinical benefit group but was significantly decreased in the non-clinical benefit group. The diversity of TCR clones with high abundance contributing to 50% of the samples was also related to clinical efficacy. The higher the D50 index, the more likely the patients were to show durable benefit. A significant difference in PFS rate was identified between the D50 high and D50 low groups. We validated this result in an independent cohort of NSCLC patients treated with anti-PD-1/PD-L1 (N = 11). At a time point of six weeks, the D50 index could differentiate patients with durable benefit. D50 at baseline was also found to be associated with survival and response in patients treated with anti-PD-1 in previously published studies (21–23). From the above findings, we can conclude that the diversity of TCR during treatment, especially the diversity of high-abundance TCR, could be used to dynamically monitor whether patients will show durable benefit in the future. Moreover, the D50 index is a potential marker that could predict durable clinical benefit and PFS. The Shannon–Weiner index of the TCR clone with frequency higher than 0.1% at baseline and the D50 index during therapy together proved that only the dominant TCR clone accounts for clinical outcomes.

The sRLs are regulators of over-inflammation and immune response, indicating their potential role as therapeutic markers of immunotherapy. Therefore, we further explored the role of sRLs in peripheral blood combined with the TCR repertoire in predicting the efficacy of ICIs. Expression of sCD27 in peripheral blood at baseline and its changes during treatment were both found to be related to the efficacy of ICIs. CD27 acts as a co-stimulatory molecule involved in the activation and proliferation of T cells and plays an important part in the function of T cells (24). Initial CD4+ and CD8+ T cell activation upregulates CD27 expression, which leads to the release of CD27 from the surfaces of activated T cells, resulting in circulating soluble CD27 (sCD27) (25). However, persistent triggering of CD27, such as that caused by the constitutive expression of CD70 in transgenic mice, leads to progressive and ultimately fatal defects of T cells, natural killer cells, and B cells, caused by direct exhaustion and activation-induced cell death or IFN-γ-mediated indirect depletion. Therefore, CD27 signaling can either improve the function of effector T cells or enhance their exhaustion and death. In this study, patients with higher expression of sCD27 showed higher DCB rates, whereas those with significantly upregulated sCD27 during treatment had lower DCB rates. A combination of anti-PD-1/PD-L1 and an anti-CD27 agonist was recently examined in clinical trials. To obtain the best clinical results with such a combination, it may be necessary to consider the intensity, timing, and duration of treatment.

The role of these tumor immune-related factors in tumor immune regulation remains to be further studied. Analysis of sRLs revealed that cytokines with similar functions showed similar expression trends. In this study, we introduced the concept of a signature and found different fold-change expression patterns of sRL signatures between baseline and post-treatment samples. Signature-1 could serve as a negative predictive factor, as its expression was significantly higher in the NDB group than in the DCB group. Interestingly, Signature-1 mainly contained immune checkpoint proteins. After subdivision into co-inhibition and co-stimulation protein groups, the two subgroups of proteins also showed significant differences between the NDB and DCB groups. We validated the clinical prediction efficacy of Signature-1 in three independent cohorts from previous studies. We also found that Signature-1 was associated with intratumoral immune compartments, including CD4+ and CD8+ T cells and PD-L1 expression. These results indicate that the signature, as a global indicator of integration of multiple functionally similar molecules, is better able to predict ICI efficacy than a single molecule.

Owing to the limited sample size, some of our results did not reach statistical significance and lacked validation. We tried to use a combination of the TCR D50 index and sRL signature to predict DCB and PFS. The DCB rate was significantly higher in the D50 high and signature low groups (Fisher’s exact test, p = 0.0089), whereas the PFS rate did not show a significant difference (**Supplementary Figure 4B**). However, these results are meaningful as preliminary findings. Besides, our cohort did not include patients receiving dual-immune combination treatment, such as nivolumab plus ipilimumab, so it was not possible to directly evaluate the association between dual-immune combination treatment and the newly discovered blood biomarkers. Therefore, in our next study, we will collect more samples and consider more treatments to evaluate and explore the mechanisms underlying the existing findings. Another limitation was that the T cells sequenced in this study were unsorted. Some previous studies used sorted TCRs from CD8+ T cells (20) or CD8+PD-1+TCR (26) to evaluate diversity and found that TCR diversity had predictive value. Unsorted TCR information may be affected by other non-tumor-related TCRs, resulting in biased results. We will use sorted CD8+ T cells in the future.

In conclusion, this study revealed the dynamic changes of peripheral blood biomarkers—the TCR β-chain repertoire and sRLs—before and during treatment, in patients with solid tumors treated with anti-PD-1/PD-L1. We found that diversity of the TCR dominant clone was correlated with DCB. Patients with DCB had increased overall TCR diversity and maintained the expression of the top 10 high-abundance TCRs. The D50 index of the sample during treatment could reflect the patient’s response (or lack of response), indicating that it might be useful for prognostic prediction. Upregulation of the sICP signature during treatment might serve as a negative biomarker for prediction of DCB. Moreover, sCD27 may have different roles in different DCB groups. In addition, the diversity of the TCR repertoire was correlated with the expression of sICPs, which might be directly or indirectly related to the immune changes induced by anti-PD-1/PD-L1 therapy. Further integration of the peripheral blood TCR repertoire and sICPs might improve our ability to predict the efficacy of anti-PD-1/PD-L1 therapy.

## Data Availability Statement

The original contributions presented in the study are included in the article/**Supplementary Material**. Further inquiries can be directed to the corresponding authors.

## Ethics Statement

The studies involving human participants were reviewed and approved by The Ethics Committee of the General Hospital of the People’s Liberation Army. The patients/participants provided their written informed consent to participate in this study

## Author Contributions

YL and JW contribute equally to this work. YL and JW designed and performed experiments and manuscript preparation. SJ and SS were responsible for the provision of study resources, materials, and patient access. LW and XZ performed the experiments. GZ collected samples and clinical information. JW and XL analyzed the sequencing data. YL and JW wrote the manuscript. JW, SX and XL revised the manuscript. All authors contributed to the article and approved the submitted version.

## Conflict of Interest

Authors JW, SX, and XL were employed by YuceBio Technology Company.

The remaining authors declare that the research was conducted in the absence of any commercial or financial relationships that could be construed as a potential conflict of interest.
