# Supplementary material for: Diversity of Dominant Peripheral T Cell Receptor Clone and Soluble Immune Checkpoint Proteins Associated With Clinical Outcomes Following Immune Checkpoint Inhibitor Treatment in Advanced Cancers
Source: Front Immunol. 2021 Jun 7;12:649343. doi: 10.3389/fimmu.2021.649343 (PMC8216394; doi:10.3389/fimmu.2021.649343)
Supplement: Supplementary file 1 [file DataSheet_1.docx]

Supplementary Material

# Supplementary Tables

**Supplementary Table 1.** Detailed clinical data of 31 patients with solid tumor.

| sample id | patient id | **sex** | **age** | response | cancer | weeks | immune drug | therapy | **lines of treatment** |
| --- | --- | --- | --- | --- | --- | --- | --- | --- | --- |
| S01 | 01 | male | 71 | - | NSCLC | 0 | Pembrolizumab+Atezolizumab | combination | second |
| S02 | 01 |  |  | SD | NSCLC | 6 | Pembrolizumab+Atezolizumab | combination | second |
| S03 | 01 |  |  | SD | NSCLC | 15 | Pembrolizumab+Atezolizumab | combination | second |
| S04 | 02 | male | 64 | - | NSCLC | 0 | Atezolizumab | combination | first |
| S05 | 02 |  |  | PR | NSCLC | 6 | Atezolizumab | combination | first |
| S06 | 02 |  |  | SD | NSCLC | 9 | Atezolizumab | combination | first |
| S07 | 02 |  |  | SD | NSCLC | 15 | Atezolizumab | combination | first |
| S08 | 03 | female | 34 | - | NSCLC | 0 | Nivolumab | single-agent | second |
| S09 | 03 |  |  | SD | NSCLC | 15 | Nivolumab | single-agent | second |
| S10 | 04 | male | 58 | - | NSCLC | 0 | Pembrolizumab | combination | multi |
| S11 | 04 |  |  | PD | NSCLC | 3 | Pembrolizumab | combination | multi |
| S12 | 04 |  |  | PD | NSCLC | 6 | Pembrolizumab | combination | multi |
| S13 | 05 | female | 67 | - | NSCLC | 0 | Pembrolizumab | combination | second |
| S14 | 05 |  |  | SD | NSCLC | 3 | Pembrolizumab | combination | second |
| S15 | 06 | male | 58 | - | UC | 0 | Pembrolizumab | combination | first |
| S16 | 06 |  |  | PD | UC | 3 | Pembrolizumab | combination | first |
| S17 | 07 | female | 52 | - | GC | 0 | Nivolumab | combination | third |
| S18 | 07 |  |  | PR | GC | 3 | Nivolumab | combination | third |
| S19 | 08 | female | 38 | - | GC | 0 | Nivolumab | combination | first |
| S20 | 08 |  |  | PD | GC | 3 | Nivolumab | combination | first |
| S21 | 09 | male | 79 | - | NSCLC | 0 | Nivolumab | single-agent | first |
| S22 | 12 | male | 45 | - | NSCLC | 0 | Nivolumab | combination | third |
| S23 | 12 |  |  | SD | NSCLC | 3 | Nivolumab | combination | third |
| S24 | 13 | female | 33 | - | NSCLC | 0 | Atezolizumab | combination | first |
| S25 | 13 |  |  | PR | NSCLC | 6 | Atezolizumab | combination | first |
| S26 | 13 |  |  | PR | NSCLC | 12 | Atezolizumab | combination | first |
| S27 | 13 |  |  | SD | NSCLC | 15 | Atezolizumab | combination | first |
| S28 | 14 | male | 59 | - | HCC | 0 | Nivolumab | single-agent | third |
| S29 | 14 |  |  | SD | HCC | 3 | Nivolumab | single-agent | third |
| S30 | 14 |  |  | SD | HCC | 6 | Nivolumab | single-agent | third |
| S31 | 11 | female | 33 | - | NSCLC | 0 | Nivolumab | combination | multi |
| S32 | 11 |  |  | SD | NSCLC | 6 | Nivolumab | combination | multi |
| S33 | 11 |  |  | SD | NSCLC | 12 | Nivolumab | combination | multi |
| S34 | 11 |  |  | SD | NSCLC | 15 | Nivolumab | combination | multi |
| S35 | 10 | male | 65 | - | NSCLC | 0 | Durvalumab | single-agent | first |
| S36 | 10 |  |  | PD | NSCLC | 12 | Durvalumab | single-agent | first |
| S37 | 16 | male | 60 | - | NSCLC | 0 | Sintilimab | single-agent | second |
| S38 | 15 | female | 61 | - | NSCLC | 0 | Pembrolizumab | single-agent | first |
| S39 | 15 |  |  | SD | NSCLC | 9 | Pembrolizumab | single-agent | first |
| S40 | 18 | male | 87 | - | UC | 0 | Atezolizumab | combination | first |
| S41 | 18 |  |  | SD | UC | 6 | Atezolizumab | combination | first |
| S42 | 18 |  |  | SD | UC | 12 | Atezolizumab | combination | first |
| S43 | 17 | female | 41 | - | NSCLC | 0 | Pembrolizumab | combination | multi |
| S44 | 17 |  |  | PD | NSCLC | 6 | Pembrolizumab | combination | multi |
| S45 | 17 |  |  | PD | NSCLC | 15 | Pembrolizumab | combination | multi |
| S46 | 19 | male | 71 | - | NSCLC | 0 | Pembrolizumab | single-agent | second |
| S47 | 19 |  |  | SD | NSCLC | 6 | Pembrolizumab | single-agent | second |
| S48 | 19 |  |  | SD | NSCLC | 12 | Pembrolizumab | single-agent | second |
| S49 | 20 | male | 59 | - | NSCLC | 0 | Pembrolizumab | single-agent | second |
| S50 | 20 |  |  | PR | NSCLC | 9 | Pembrolizumab | single-agent | second |
| S51 | 20 |  |  | PD | NSCLC | 21 | Pembrolizumab | single-agent | second |
| S52 | 21 | male | 78 | - | NSCLC | 0 | Nivolumab | single-agent | first |
| S53 | 22 | male | 66 | - | NSCLC | 0 | Nivolumab | combination | first |
| S54 | 23 | male | 77 | - | UC | 0 | Nivolumab | single-agent | second |
| S55 | 23 |  |  | SD | UC | 12 | Nivolumab | single-agent | second |
| S56 | 24 | male | 60 | - | HCC | 0 | Nivolumab | single-agent | first |
| S57 | 25 | male | 38 | - | NSCLC | 0 | Pembrolizumab | combination | second |
| S58 | 25 |  |  | PD | NSCLC | 9 | Pembrolizumab | combination | second |
| S59 | 26 | male | 46 | - | UC | 0 | Pembrolizumab | combination | first |
| S60 | 26 |  |  | SD | UC | 3 | Pembrolizumab | combination | first |
| S61 | 26 |  |  | SD | UC | 12 | Pembrolizumab | combination | first |
| S62 | 27 | male | 50 | - | NSCLC | 0 | Nivolumab | single-agent | second |
| S63 | 27 |  |  | PD | NSCLC | 18 | Nivolumab | single-agent | second |
| S64 | 29 | female | 35 | - | UC | 0 | Nivolumab | single-agent | second |
| S65 | 29 |  |  | PR | UC | 18 | Nivolumab | single-agent | second |
| S66 | 31 | female | 40 | - | NSCLC | 0 | Pembrolizumab | combination | first |
| S67 | 31 |  |  | PR | NSCLC | 6 | Pembrolizumab | combination | first |
| S68 | 28 | male | 64 | - | ICC | 0 | Pembrolizumab | combination | third_and_multi |
| S69 | 28 |  |  | PD | ICC | 15 | Pembrolizumab | combination | third_and_multi |
| S70 | 28 |  |  | PR | ICC | 21 | Pembrolizumab | combination | third_and_multi |
| S71 | 30 | male | 69 | - | NSCLC | 0 | Pembrolizumab | single-agent | second |
| S72 | 30 |  |  | SD | NSCLC | 36 | Pembrolizumab | single-agent | second |

**Supplementary Table 2.** Shared significantly expanded TCR clone.

| **Patient ID** | **Clone** | **Frequency of baseline** | **Frequency of post-treatment** | **P-value** | **Q-value** |
| --- | --- | --- | --- | --- | --- |
|  |  |  |  |  |  |
| 23 | CASSSGTYGGGAADTQYF | <0.0001 | 0.0012 | 1.63E-59 | 2.53E-57 |
| 28 | CASSSGTYGGGAADTQYF | 0.0528 | 0.2048 | 0.00E+00 | 0.00E+00 |
| 23 | CASSPRWTGPTGGTEAFF | <0.0001 | 0.0011 | 8.26E-55 | 1.10E-52 |
| 28 | CASSPRWTGPTGGTEAFF | 0.0315 | 0.0874 | 0.00E+00 | 0.00E+00 |
| 01 | CASSLQVNTEAFF | 0.0011 | 0.0001 | 9.99E-68 | 8.78E-66 |
| 11 | CASSLQVNTEAFF | 0.0101 | 0.0219 | 6.22E-211 | 2.67E-208 |

**Supplementary Table 3.** Detailed clinical data of 12 patients with solid tumor in validation cohort.

| patient id | sex | response | immune drug | cancer | lines of treatment | therapy |
| --- | --- | --- | --- | --- | --- | --- |
| V01 | male | SD | Nivolumab | NSCLC | second | single-agent |
| V02 | male | PR | Nivolumab | NSCLC | second | single-agent |
| V03 | male | SD | Nivolumab | NSCLC | second | single-agent |
| V04 | male | PR | Nivolumab | NSCLC | second | single-agent |
| V05 | male | PD | Nivolumab | NSCLC | second | single-agent |
| V06 | female | PD | Nivolumab | NSCLC | second | single-agent |
| V07 | male | PD | Pembrolizumab | NSCLC | multi | combine |
| V08 | male | SD | Nivolumab | NSCLC | first | combine |
| V09 | male | SD | Atezolizumab | NSCLC | first | combine |
| V10 | male | SD | Nivolumab | NSCLC | third | single-agent |
| V11 | male | PD | Sintilimab | NSCLC | second | single-agent |
| V12 | female | PD | Nivolumab | NSCLC | second | single-agent |

# Supplementary Figures


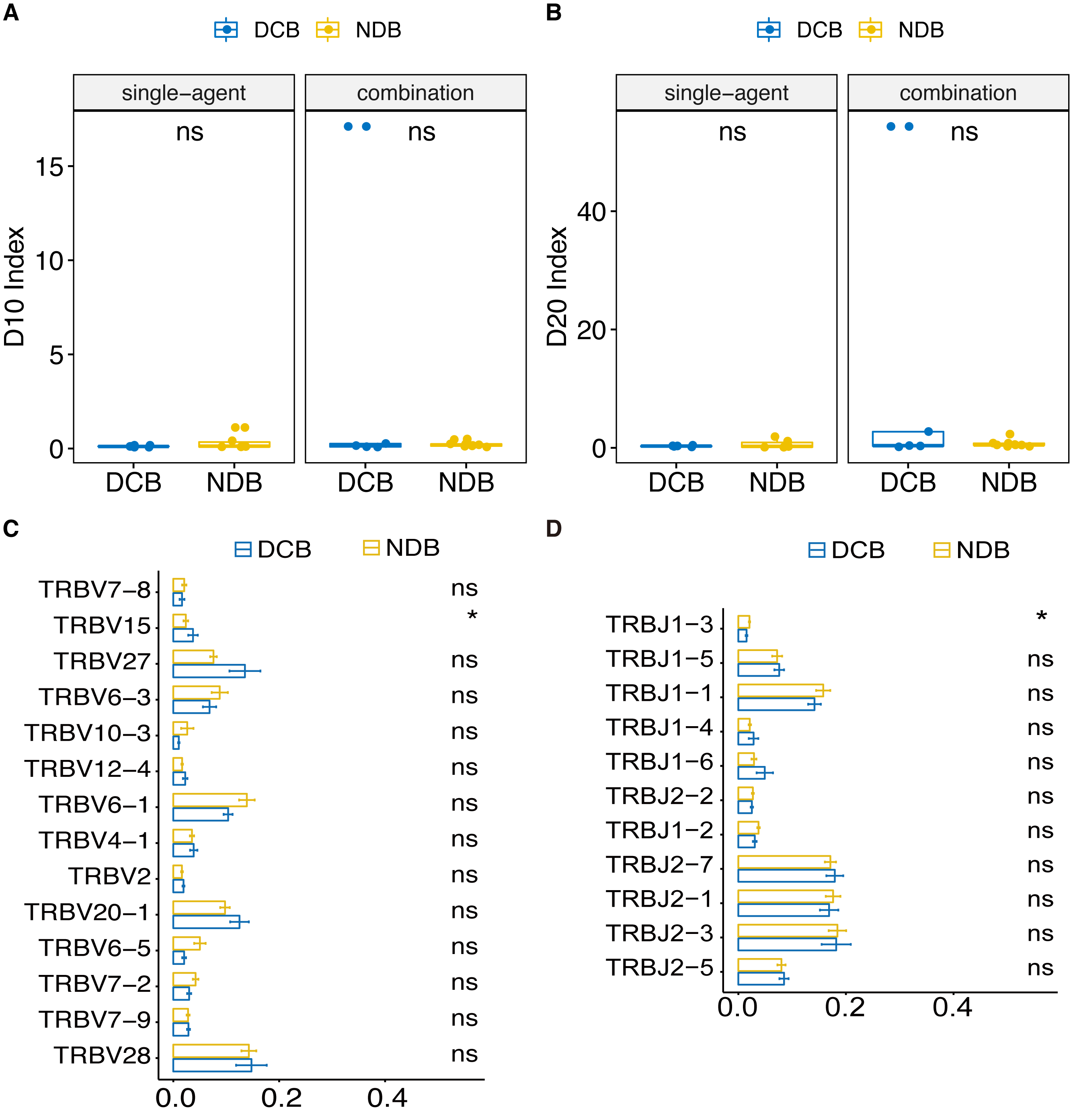


**Supplementary Figure 1.** **Association between index and durable clinical benefit and the usage of Vβ, Jβ, and Vβ-Jβ paired genes in samples.** (A) Association between D10 index and durable clinical benefit. (B) Association between D20 index and durable clinical benefit. The usage frequency difference in Vβ/Jβ genes between DCB and NDB group in baseline group samples. Compared with the NDB group, there was no significant change in the frequency of the V gene in the DCB group (C), while the TRJ1-3 / TRJ1-2 cloning frequency was down-regulated in the J gene (D). *P < .05 and **P < .01 by two-tailed paired t-tests.


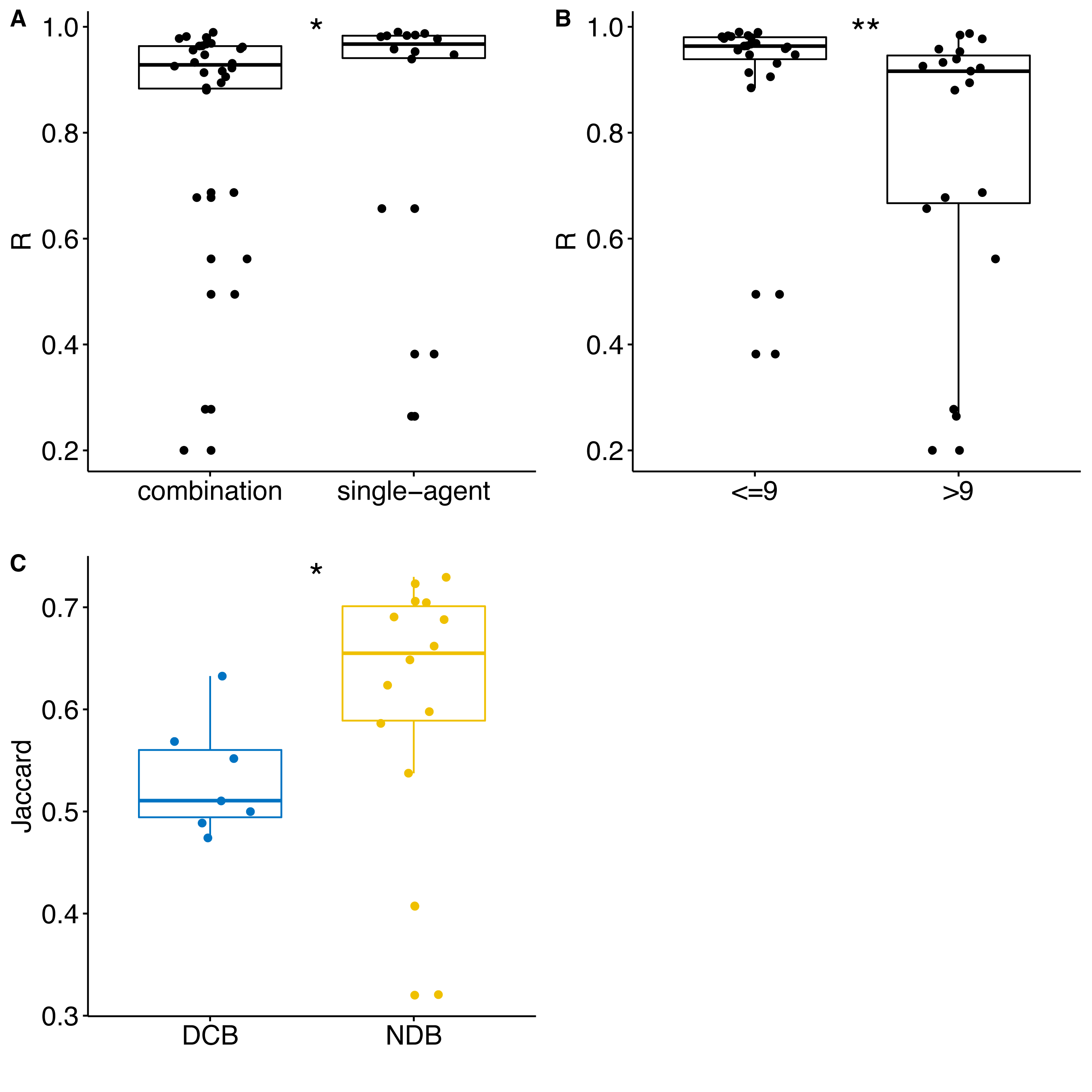


**Supplementary Figure 2. Comparsion on similarity of TCR repertoire between pre- and post- treatment samples with treatment time, therapy and clinical benefit groups.** Similarity index R was calculated by paired person correlation. Similarity index Jaccard was calculated by determining what clones were present in both pre- and post- treatment samples. Statistical analysis was performed using the Mann-Whitney test. Boxes depict the interquartile range with the line in boxes show the median, and the lines outside the boxes show the first or third quartiles of fraction. ns p >= 0.05, *p < 0.05, **p < 0.01. (A) Similarity comparsion between patient with second blood sample collected within 9 weeks and longer than 9 weeks. (B) Similarity comparsion between patient treated with combination and single-agent. (C) Similarity comparsion between clinical benefit (DCB) patients and non- clinical benefit (NDB) patients in patients treated with combination.


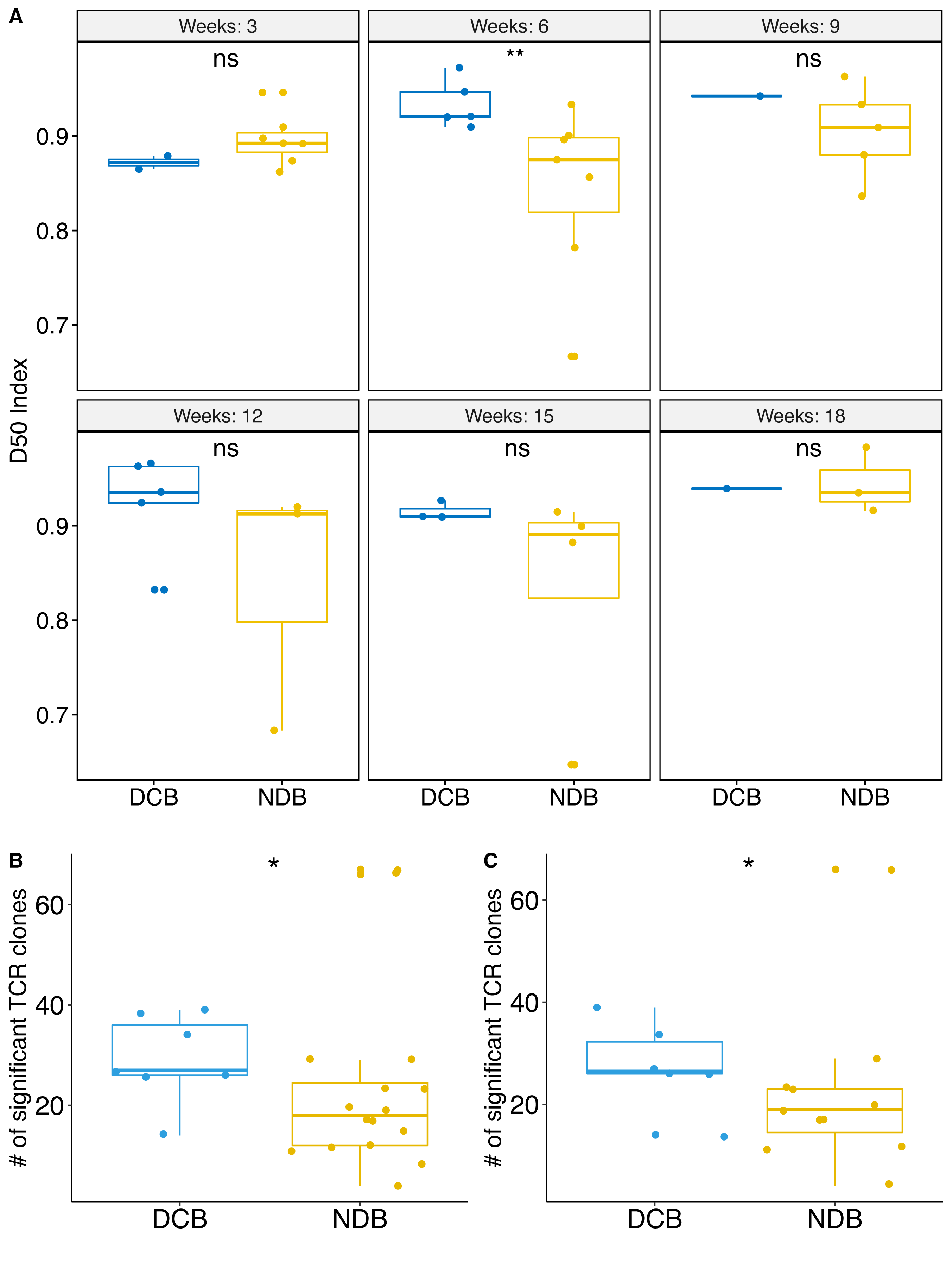


**Supplementary Figure 3. TCR dynamic dynamic change during treatment.** (A) Comparsion on D50 index of TCR repertoire at post-treatment samples in different time point with clinical benefit groups. (B) Significant differential expressed clones identified from pre- to post- treatment samples.in clinical benefit (DCB) patients and non- clinical benefit (NDB) patients in all patient cohort. (C) Significant differential expressed clones identified from pre- to post- treatment samples.in clinical benefit (DCB) patients and non- clinical benefit (NDB) patients in combination treatment patient cohort. Statistical analysis was performed using the Mann-Whitney test. Boxes depict the interquartile range with the line in boxes show the median, and the lines outside the boxes show the first or third quartiles of fraction. ns p >= 0.05, *p < 0.05, **p < 0.01.


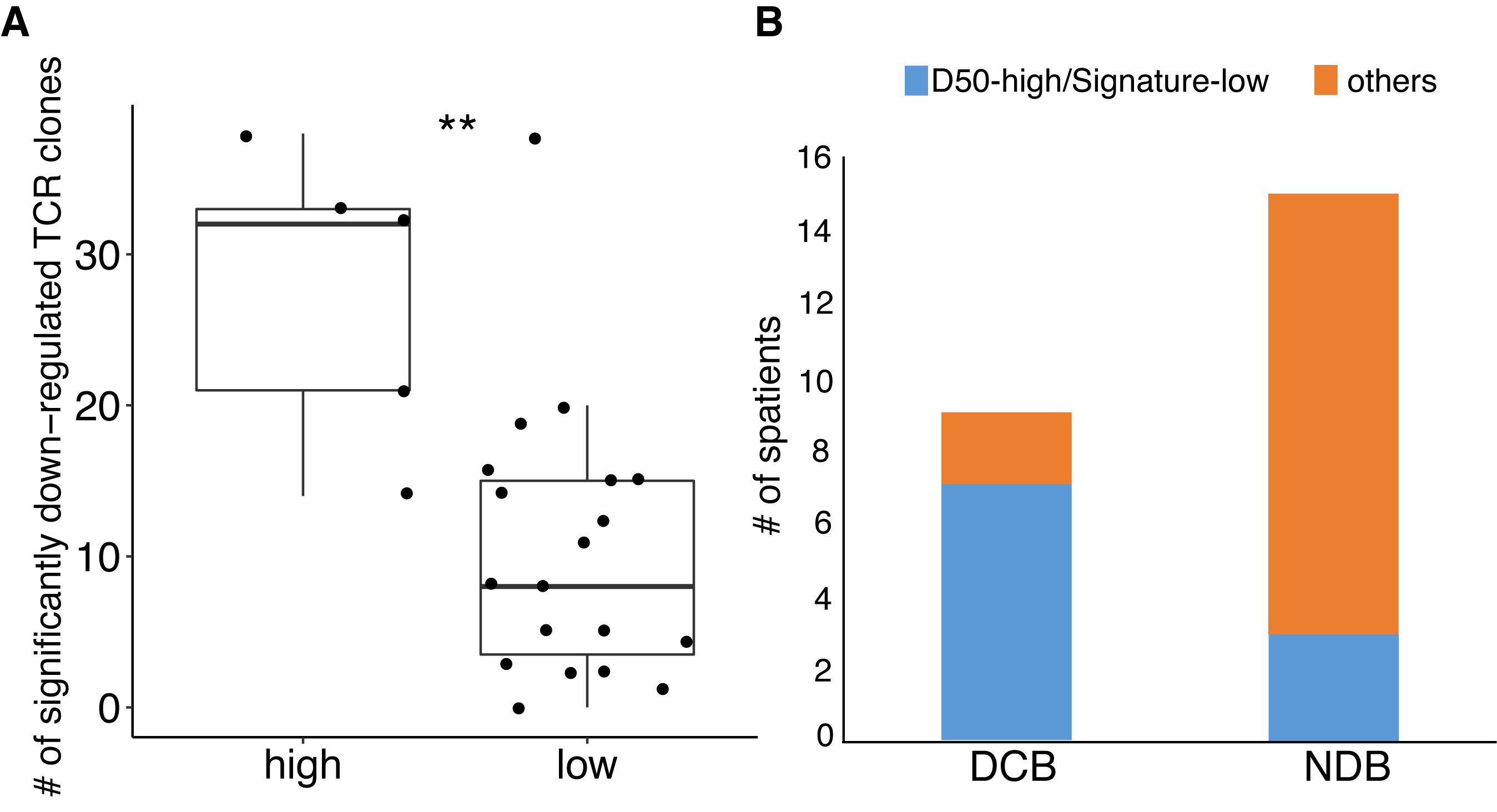


**Supplementary Figure 4. Association and combination of TCR change and soluble Signature-1** (A) Comparsion on number of significant down-regulated TCR clone between high regulated and low-regulated expression of soluble immune checkpoint proteins (Signature-1). Boxes depict the interquartile range with the line in boxes show the median, and the lines outside the boxes show the first or third quartiles of fraction. ns p >= 0.05, *p < 0.05, **p < 0.01. (B) Correlations between combined indicator and durable clinical benefit. Fisher’s exact one-sided test was used for statistic, p value is 0.067.


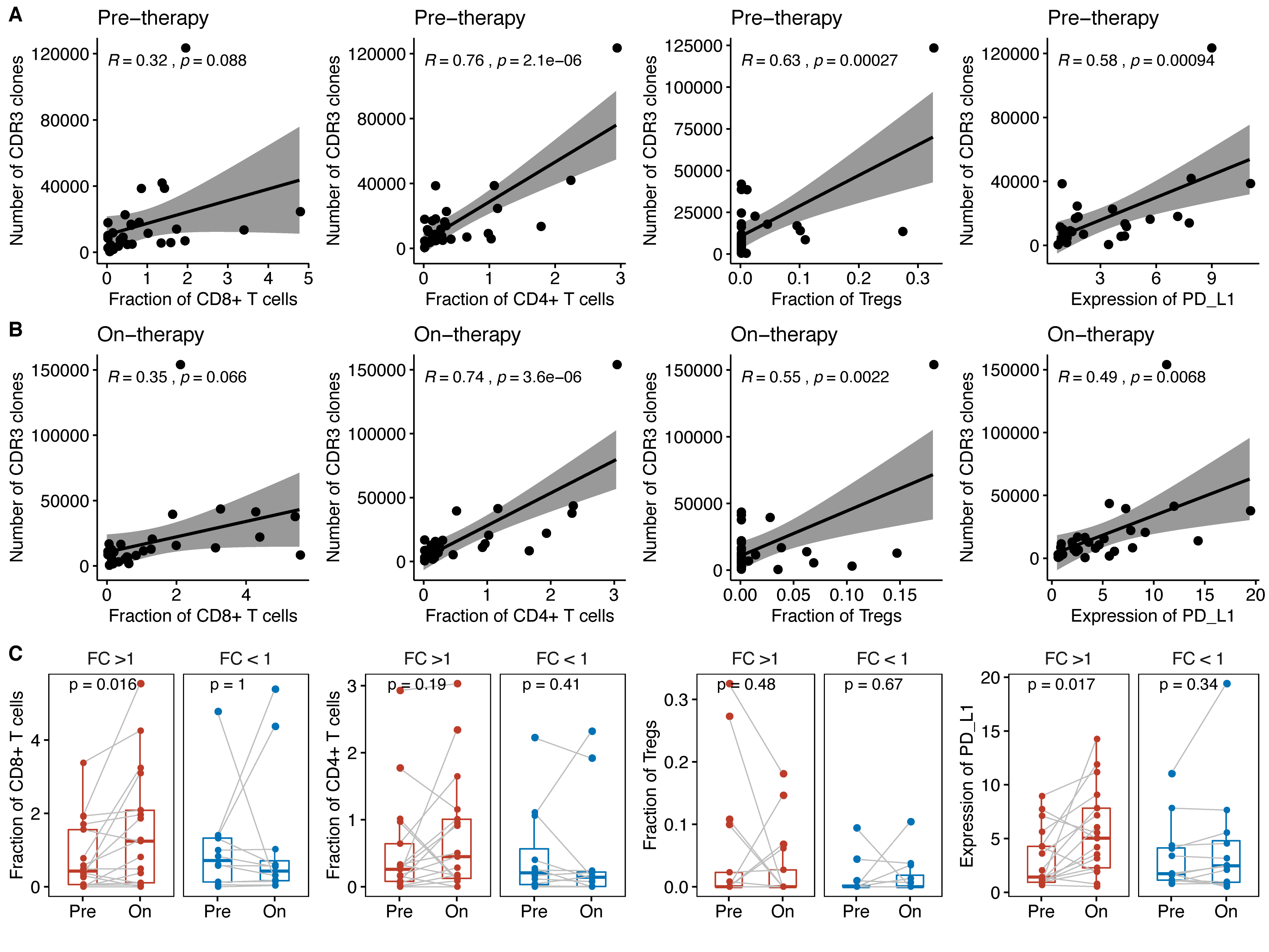


**Supplementary Figure 5. Correlations between intratumoral immune compartment and TCR clonality in three published cohorts.** Correlations between fraction of CD8+ T cells, CD4+ T cells, Tregs and PD-L1 expression level and TCR clonality in pre-therapy (baseline) (A) and on-therapy samples (B). (C) Dynamic change of fraction of CD8+ T cells, CD4+ T cells, Tregs and PD-L1 expression level between pre-therapy (baseline) and on-therapy samples in TCR clonality “FC > 1” and “FC < 1” groups. FC > 1: fold change of TCR clonality in on-therapy and pre-therapy samples > 1; FC < 1: fold change of TCR clonality in on-therapy and pre-therapy samples < 1.
